# The Annotated Genome of Wolbachia from the Filarial Nematode Brugia malayi: What It Means for Progress in Antifilarial Medicine

**DOI:** 10.1371/journal.pmed.0020110

**Published:** 2005-04-26

**Authors:** Kenneth Pfarr, Achim Hoerauf

## Abstract

Filarial nematodes contain endosymbiotic bacteria of the genus *Wolbachia.* As described in the April 2005 issue of *PLoS Biology,* Foster et al. have sequenced the genome of the *Wolbachia* that lives in the nematode *Brugia malayi.* What are the clinical implications?

## Filariasis

The filarial nematodes Wuchereria bancrofti, Brugia spp., and Onchocerca volvulus are insect-borne parasites that cause lymphatic or cutaneous filariasis. Lymphangitis, hydrocele, and elephantiasis are pathologies that result from W. bancrofti and Brugia spp. infections. O. volvulus infections can present with severe skin pathologies (acute and chronic dermatitis, atrophy) and blindness (onchocerciasis, or river blindness). The nematodes infect more than 140 million people in 90 mostly tropical countries. An additional one billion people are at risk of contracting the diseases caused by these nematodes [1,2].

Current control efforts, both vector control and mass antifilarial chemotherapies, have shown initial success, but sustainability is uncertain. For example, vector control, used for onchocerciasis in west African savannah areas, has become too expensive and therefore been stopped. Current and planned elimination programs will rely on mass administration of antifilarial drugs that require annual administration for 5–10 years for lymphatic filariasis and more than 20 years for onchocerciasis.

Control programs based on drug administration require long treatment durations because the adult female worms, which produce thousands of larvae daily, survive many years (over 14 years for onchocerciasis) and are not killed by current drugs [1]. The success of such control programs, as shown by mathematical modeling, depends on a minimum of 60% of the people in an endemic area participating every year [3]. This is probably too optimistic an estimate, as a recent review of onchocerciasis therapy in regions that have had 10–12 years of ivermectin treatment still show infection levels of 2%–3%. These levels are enough to establish the infection within a few years after the end of annual drug administration [4]. Additionally, there is evidence that some geographic areas have worms with apparent resistance to ivermectin [5]. Therefore, it is essential that we find new drugs that kill or sterilize adult worms.

## Enter Wolbachia—Endosymbionts of Filarial Nematodes

**Figure 1:**
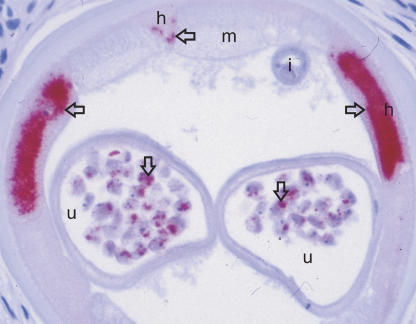
Cross-Section of a Female Worm from an Extirpated Nodule from a Patient with Onchocerciasis Wolbachia, endosymbiotic bacteria of filarial nematodes important for embryo development, are targets for antifilarial treatment. The endobacteria cause inflammation and contribute to the pathology of lymphatic filariasis and onchocerciasis. The bacteria here were stained with antibodies against Yersinia Hsp60. The bacteria are located in the hypodermis and oocytes of the worm (arrows). h, hypodermis; i, intestine; m, muscle; u, uterine tube. (Photo: D.W. Büttner, Bernhard Nocht Institute for Tropical Medicine, Hamburg, Germany)

For almost 30 years, it has been known that filarial nematodes contain endosymbiotic bacteria. These endobacteria are found in the hypodermis of male and female worms, and in the oocytes, embryos, and larval stages (Figure 1). As in many animal filarial species, endobacteria are present in the human filariae W. bancrofti, Brugia spp., and O. volvulus [1,6,7], but not in Loa loa [8,9]. Recently, these endosymbionts were classified at the molecular level to be of the genus Wolbachia, a genus of bacteria that are common endosymbionts of arthropods. The next nearest relatives of Wolbachia are Rickettsia, Ehrlichia, Cowdria, and Anaplasma [10].

Studies of the effect of antirickettsials such as tetracycline and rifampicin in animals infected with filarial nematodes have shown, using immunohistochemistry, that these drugs deplete the Wolbachia from the worms. After the Wolbachia are depleted, the worms develop a distinct phenotype. Monitoring the microfilaria (Mf) levels in the blood showed that the number of Mf in the treated animals was lower than the number in the control groups, and that the number of Mf in the treated group neared zero with time. Examination of the adult worms showed that embryogenesis was blocked and the uteri contained degenerated embryos [1]. A study done in cattle infected with O. ochengi even showed killing of adult worms [11].

The success of antibiotic treatment against animal filariae has been extended to human filarial infections. Trials of doxycycline have been completed for populations infected with O. volvulus and W. bancrofti. For both species, larval levels were zero or near zero after treatment [6,12]. Examination of worms from extirpated nodules of patients with onchocerciasis showed that, as in the animal studies, depletion of Wolbachia led to a block in embryogenesis that appears to be permanent [2]. Most recently, there has been evidence for the killing of adult W. bancrofti by doxycycline therapy [13].

Wolbachia stimulate inflammatory responses via Toll-like receptor (TLR) 2 and TLR4 [14], pattern recognition receptors that recognize a variety of bacterial molecules [15]. Working with a mouse model of O. volvulus–induced corneal inflammation (keratitis), it has been shown that Wolbachia antigens presented via TLR4 are required for the development of pathology [16]. Wolbachia have also been associated with adverse reactions seen in infected patients after antifilarial therapy. Recent studies supporting a role for Wolbachia in adverse reactions after antifilarial treatment have shown that doxycycline given before ivermectin reduced Wolbachia loads as well as the number and severity of adverse reactions in patients with lymphatic filariasis ([17]; J. Turner, S. Mand, A. Y. Debrah, J. Muehlfeld J, K. M. Pfarr, et al., unpublished data). Thus, doxycycline fulfills the role for a new antifilarial therapy in that it produces sterility in lymphatic filariasis and onchocerciasis, kills adult worms in lymphatic filariasis, and prevents or lessens adverse reactions due to the rapid killing of Mf by microfilaricidal drugs. However, the treatment time of four weeks is still longer than that desired for new antifilarial therapies. Wolbachia are ideal targets for antifilarial drugs that have the same effect as doxycycline, but that work in a shorter interval. Potential drug targets may be found by analyzing the genome of Wolbachia.

## Sequencing the Wolbachia Genome

**Figure 2:**
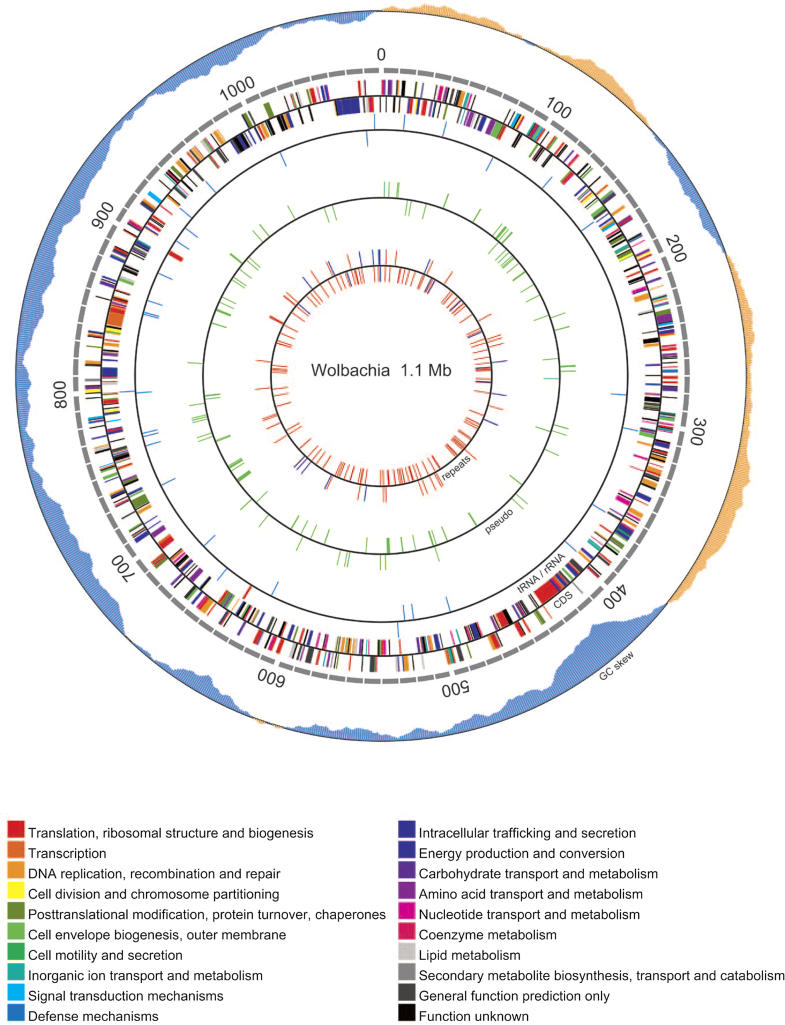
Annotation of the Complete Wolbachia Genome (Figure from [18])

As part of the effort to find antiwolbachial drugs that act in less time than the current four-week regime for doxycycline, a consortium was established to sequence the genomes of the Wolbachia species that inhabit human filarial nematodes. In the April 2005 issue of *PLoS Biology*, Foster and colleagues report on the completion of the sequencing and annotation of the genome from the Wolbachia of Brugia malayi (*w*Bm) (Figure 2) [18]. This is the first complete Wolbachia genome from a filarial nematode. The authors compare the *w*Bm genome to the first sequenced genome of the Wolbachia of Drosophila melanogaster (*w*Mel), which is strictly parasitic, and the genomes of other endosymbiotic bacteria, pointing out potential metabolites that *w*Bm may supply to the nematode.

## Features of the Genome and Metabolites that *w*Bm May Provide to Its Host

The genome of *w*Bm is 1.1 million nucleotides, smaller than *w*Mel and Rickettsia prowazekii, but larger than that of Mycobacterium. The reduced genome size is indicative of long-term symbiosis, and reflects the loss of all genes necessary to make all but one amino acid, genes needed to infect new hosts, and genes needed to evade the host immune system. Also lacking from the *w*Bm genome are several genes needed for DNA repair and genes required for RNA modification. Many of these genes have also been lost from other endosymbiotic bacteria. *w*Bm cannot synthesize lipopolysaccharide, a component of the cell membrane in most bacteria. This is astounding because, as noted above, Wolbachia stimulate an inflammatory reaction via TLR4. Endobacteria antigens presented via TLR4 are also responsible for adverse reactions after antifilarial treatment [17], and for pathology in onchocerciasis [16]. *w*Bm lack the genes necessary to cross-link and degrade the carbohydrate backbone of peptidoglycan. The Wolbachia peptidoglycan structure resembles the peptidoglycan-derived cytotoxins produced by Neisseria gonorrhoeae and Bordetella pertussis, which are made up of muramyl peptides [19]. These muramyl peptides are known to stimulate an inflammatory response and pathology via TLR2.

The completion of the wBm genome offers a wealth of information for both basic and applied science.

However, to understand the Wolbachia–filaria endosymbiosis, it is not only important what *w*Bm has lost from the genome during its long symbiosis with B. malayi, but also what has been kept. The endobacteria have retained all of the genes necessary for the synthesis of the co-factors riboflavin, flavin adenine dinucleotide, and heme. The genome also contains the genes necessary to make glutathione, although it lacks the genes needed for glutathione-mediated methylglyoxal detoxification [20], which is the most common reason intracellular bacteria need glutathione. Finally, in contrast to most endosymbiotic bacteria, wBm have retained the genes necessary to make all nucleotides.

As the *w*Bm genome has the genes for a type IV secretion system, used by intracellular bacteria for exporting molecules to nonbacterial (e.g., host) cells, the above described metabolites could be supplied to the nematode host by Wolbachia. To date, there is no evidence of genes for riboflavin and heme synthesis in the B. malayi genome [21]. Heme from Wolbachia could be vital to worm embryogenesis, as there is evidence that molting and reproduction are controlled by ecdysteroid-like homones [22], whose synthesis requires heme. Depletion of Wolbachia would therefore halt production of these hormones and block embryogenesis. Wolbachia could be a source of glutathione which, aside from its role in the detoxification of methylglyoxal, is required for protection against oxidative stress from oxygen-reactive species secreted by mammalian immune cells [23]. Finally, Wolbachia may be an essential source of nucleotides during embryogenesis. Wolbachia as a source of the above metabolites would explain the block in embryogenesis and the sterility seen in worms after depletion of the endobacteria.

## Conclusion

The completion of the *w*Bm genome offers a wealth of information for both basic and applied science. With the completion of this genome, one can now compare close relatives that infect different hosts and have different symbiotic lifestyles, i.e., parasitic versus mutualistic. Such a comparison of the differences could help to define genes necessary for invading host cells. Examining the genome of *w*Bm will help us understand the molecular basis for the endosymbiosis between filarial nematodes and Wolbachia. Researchers now know which metabolites the endobacteria require from the nematode host and which might be provided by Wolbachia to the nematode. This is exciting because it opens up the possibility to find and test drugs already registered for use in humans that might inhibit key biochemical pathways in the Wolbachia—and lead to sterility or killing of the adult worms in shorter treatment times—and that could be given to all infected persons. Given the huge challenges that still lie ahead for the programs to eliminate filariasis, such a need has been and will be repeatedly stated. The sequencing and annotation of the wBm genome will aid researchers in fulfilling this goal by focusing research on those biochemical events that are essential to the mutualistic symbiosis between filarial nematodes and their Wolbachia.

## Abbreviations

Mf: microfilaria or microfilariae
TLR: Toll-like receptor
*w*Bm: Wolbachia of Brugia malayi
*w*Mel: Wolbachia of Drosophila melanogaster

## References

[pmed-0020110-b1] Molyneux DH, Bradley M, Hoerauf A, Kyelem D, Taylor MJ (2003). Mass drug treatment for lymphatic filariasis and onchocerciasis. Trends Parasitol.

[pmed-0020110-b2] Hoerauf A, Büttner DW, Adjei O, Pearlman E (2003). Onchocerciasis. BMJ.

[pmed-0020110-b3] Michael E, Malecela-Lazaro MN, Simonsen PE, Pedersen EM, Barker G (2004). Mathematical modeling and the control of lymphatic filariasis. Lancet Infect Dis.

[pmed-0020110-b4] Borsboom GJ, Boatin BA, Nagelkerke NJ, Agoua H, Akpoboua KL (2003). Impact of ivermectin on onchocerciasis transmission: Assessing the empirical evidence that repeated ivermectin mass treatments may lead to elimination/eradication in West-Africa. Filaria J.

[pmed-0020110-b5] Awadzi K, Attah SK, Addy ET, Opoku NO, Quartey BT (2004). Thirty-month follow-up of sub-optimal responders to multiple treatments with ivermectin, in two onchocerciasis-endemic foci in Ghana. Ann Trop Med Parasitol.

[pmed-0020110-b6] Hoerauf A, Mand S, Adjei O, Fleischer B, Büttner D (2001). Depletion of Wolbachia endobacteria in Onchocerca volvulus by doxycycline and microfilaridermia after ivermectin treatment. Lancet.

[pmed-0020110-b7] Hoerauf A, Volkmann L, Hamelmann C, Adjei O, Autenrieth IB (2000). Endosymbiotic bacteria in worms as targets for a novel chemotherapy in filariasis. Lancet.

[pmed-0020110-b8] Büttner DW, Wanji S, Bazzocchi C, Bain O, Fischer P (2003). Obligatory symbiotic Wolbachia endobacteria are absent from Loa loa. Filaria J.

[pmed-0020110-b9] McGarry HF, Pfarr K, Egerton G, Hoerauf A, Akue JP (2003). Evidence against Wolbachia symbiosis in Loa loa. Filaria J.

[pmed-0020110-b10] Casiraghi M, Anderson TJC, Bandi C, Bazzocchi C, Genchi C (2001). A phylogenetic analysis of filarial nematodes: Comparison with the phylogeny of Wolbachia endosymbionts. Parasitol.

[pmed-0020110-b11] Langworthy NG, Renz A, Mackenstedt U, Henkle-Dührsen K, Bronsvoort MB (2000). Macrofilaricidal activity of tetracycline against the filarial nematode Onchocerca ochengi: Elimination of Wolbachia precedes worm death and suggests a dependent relationship. Proc R Soc Lond B Biol Sci.

[pmed-0020110-b12] Hoerauf A, Mand S, Fischer K, Kruppa T, Marfo-Debrekyei Y (2003). Doxycycline as a novel strategy against bancroftian filariasis-depletion of Wolbachia endosymbionts from Wuchereria bancrofti and stop of microfilariae production. Med Microbiol Immunol.

[pmed-0020110-b13] Taylor MJ, Makunde WH, McGarry HF, Turner JD, Mand S (2004). Macrofilaricidal activity following doxycycline treatment of Wuchereria bancrofti: A double-blind randomised controlled trial. Lancet.

[pmed-0020110-b14] Brattig NW, Bazzocchi C, Kirschning CJ, Reiling N, Büttner DW (2004). The major surface protein of Wolbachia endosymbionts in filarial nematodes elicits immune responses through TLR2 and TLR4. J Immunol.

[pmed-0020110-b15] Akira S (2003). Toll-like receptor signaling. J Biol Chem.

[pmed-0020110-b16] Saint André A, Blackwell NM, Hall LR, Hoerauf A, Brattig NW (2002). The role of endosymbiotic Wolbachia bacteria in the pathogenesis of river blindness. Science.

[pmed-0020110-b17] Keiser PB, Reynolds SM, Awadzi K, Ottesen EA, Taylor MJ (2002). Bacterial endosymbionts of Onchocerca volvulus in the pathogenesis of posttreatment reactions. J Infect Dis.

[pmed-0020110-b18] Foster J, Ganatra M, Kamal I, Ware J, Makarova K (2005). The Wolbachia genome of Brugia malayi: Endosymbiont evolution within a human pathogenic nematode. PLoS Biol.

[pmed-0020110-b19] Cloud KA, Dillard JP (2002). A lytic transglycosylase of Neisseria gonorrhoeae is involved in peptidoglycan-derived cytotoxin production. Infect Immun.

[pmed-0020110-b20] Booth IR, Ferguson GP, Miller S, Li C, Gunasekera B (2003). Bacterial production of methylglyoxal: A survival strategy or death by misadventure?. Biochem Soc Trans.

[pmed-0020110-b21] Ghedin E, Wang S, Foster JM, Slatko BE (2004). First sequenced genome of a parasitic nematode. Trends Parasitol.

[pmed-0020110-b22] Warbrick EV, Barker GC, Rees HH, Howells RE (1993). The effect of invertebrate hormones and potential hormone inhibitors on the third larval moult of the filarial nematode, Dirofilaria immitis, in vitro. Parasitol.

[pmed-0020110-b23] Selkirk ME, Smith VP, Thomas GR, Gounaris K (1998). Resistance of filarial nematode parasites to oxidative stress. Int J Parasitol.

